# Fabrication of Transparent and Conductive SWCNT/SiO_2_ Composite Thin-Film by Photo-Irradiation of Molecular Precursor Films

**DOI:** 10.3390/nano11123404

**Published:** 2021-12-16

**Authors:** Naoki Ogawa, Hiroki Nagai, Yukihiro Kudoh, Takeyoshi Onuma, Taichi Murayama, Akinobu Nojima, Mitsunobu Sato

**Affiliations:** 1 Electrical Engineering and Electronics Program, Graduate School, Kogakuin University, Tokyo 192-0015, Japan; cm20014@ns.kogakuin.ac.jp (N.O.); cm21049@ns.kogakuin.ac.jp (T.M.); 2 Department of Applied Physics, School of Advanced Engineering, Kogakuin University, Tokyo 192-0015, Japan; nagai@cc.kogakuin.ac.jp (H.N.); onuma@cc.kogakuin.ac.jp (T.O.); 3 Department of Information and Communications Engineering, Faculty of Informatics, Kogakuin University, Tokyo 192-0015, Japan; kudoh@cc.kogakuin.ac.jp; 4 Advanced Products Development Center, Technology & Intellectual Property HQ, TDK Corporation, Nagano 385-8555, Japan; Akinobu.Nojima@tdk.com

**Keywords:** deep ultraviolet, vacuum ultraviolet, near infrared, transparent, conductive thin film, SWCNT/SiO_2_, molecular precursor method, scratch resistance, heat resistance

## Abstract

A single-walled carbon nanotube (SWCNT)-silica composite thin film on a quartz glass was formed by ultraviolet irradiation (20–40 °C) onto a spin-coated precursor film. With 7.4 mass% SWCNTs, the electrical resistivity reached 7.7 × 10^−3^ Ω·cm after UV-irradiation. The transmittance was >80% at 178–2600 nm, and 79%–73% at 220–352 nm. Heat treatment increased the transparency and pencil hardness, without affecting the low electrical resistivity. Raman spectroscopy and microscopic analyses revealed the excellent film morphology with good SWCNT dispersal. The low refractive index (1.49) and haze value (<1.5%) are invaluable for transparent windows for novel optoelectronic devices.

## 1. Introduction

Transparent conductive oxide (TCO) films are important for various optoelectronic devices, such as transparent electrodes for light-emitting diodes and solar cells. Transparent thin films of indium tin oxide (ITO) and fluorine tin oxide (FTO) on glass substrates are the most widely used for these purposes [1]. These common TCO thin films provide an electrical resistivity of 10^−3^ Ω·cm and a high transmittance of over 80% in the visible-light region. However, these films absorb deep ultraviolet (DUV) and vacuum ultraviolet (VUV) light because of their bandgap energies. Therefore, a new type of transparent conductive film that is adaptable over a wide spectral range, spanning VUV to near-infrared (IR) light, would spur the development of novel optoelectronic devices. The refractive index of ITO films is approximately 1.9 in the visible-light region [2], which reduces the transmittance of the electrode owing to reflections at the interface between the ITO and the glass substrate, for which the typical value is ~1.5. Thus, a transparent conductive film with a low refractive index is required.

Carbon nanotubes (CNTs) are conductive materials with excellent strength, electron conductivity, and flexibility [3]. CNTs are generally used to fabricate composite materials comprising rubber, polymers, and so on [4,5]. The CNT networks in the composites are intertwined, and electrically conductive paths are formed by contact between CNTs [6], where the conductivity depends on the diameter of the CNTs and the low resistance associated with the networks of CNTs with small diameters [7]. Therefore, SWCNTs with small diameters are suitable for highly conductive films. CNT-inorganic composites afford exceptional optical, mechanical, electrical, and thermal properties [8]. The CNT-SiO_2_ composite is considered ideal due to its transparency over a wide wavelength range and the thermal stability of silica, which arises from the good chemical interaction between the CNTs and the silica matrix [9,10]. However, it is difficult to homogeneously disperse CNTs in a silica matrix because van der Waals forces cause the CNTs to aggregate [10]. Additionally, the SWCNTs with excellent electronic properties tend to be damaged during the formation of the composite [11]. The poor dispersibility in many precursor solutions and the lack of durability during composite fabrication are still serious challenges in producing SWCNT composites.

The formation methods of thin films can be classified into dry and wet processes. The dry processes, such as sputtering, ion beam deposition and so forth. is one of the methods to obtain high-quality thin films, and generally high cost due to necessary vacuum systems. On the other hand, the wet process is generally simple with no requirement of complicated facilities such as vacuum systems, and it is applicable to forming large-area thin films. The conventional TCO thin films with low resistivity are generally formed using the dry process. However, if the TCO thin film with low resistivity can be formed by using the wet process, it is a great advantage.

Our group developed a wet chemical process, the molecular precursor method (MPM), for fabricating thin films of various metals, metal oxides, and phosphate compounds [12]. This method is based on the design of a metal complex and offers many advantages, such as excellent stability, homogeneity, miscibility, and coatability. Recently, we prepared an SiO_2_ precursor solution that is highly miscible in an aqueous dispersion of MWCNTs and used UV irradiation at ambient temperature to fabricate a thin-film of a MWCNT/SiO_2_ composite on a spin-coated precursor film [13]. The electrical resistivity and transmittance of the mechanically robust composite thin film (8H pencil hardness), were 0.7 Ω·cm and >80% in both the visible and UV regions, respectively. These results suggest that MPM can produce a SWCNT-SiO_2_ composite while preserving the excellent properties of the SWCNTs.

Herein, we report a promising composite thin film as a transparent and conductive material. The film has a thickness of 60 nm and an electrical resistivity of 10^−3^ Ω·cm. Except for the minimum transmittance of approximately 73% at approximately 273 nm in the DUV region, the film has a transparency exceeding 80% over a wide range, spanning 178 nm (VUV region) to the near IR region.

## 2. Materials and Methods

### 2.1. Materials

Tetraethyl orthosilicate (TEOS), oxalic acid, and 2-propanol were purchased from Kanto Chemical Co., Inc. (Tokyo, Japan), FUJI-FILM Wako Pure Chemical Corporation (Miyazaki, Japan), and Taisei Chemical Co., Ltd. (Tokyo, Japan). An ethanol dispersion of SWCNTs (0.2 mass%) (eDIPS-INK) was acquired from Meijo Nano Carbon Co., Ltd. (Aichi, Japan). Deionized water was purchased from Kyoei Pharmaceutical Co., Ltd. (Chiba, Japan). Ethanol (EtOH) was purchased from Ueno Chemical Industries, Ltd. (Tokyo, Japan) and dried on 4A molecular sieves prior to use. The other materials were used as received, without further purification. Polished quartz glass plates (20 × 20 × 1.5 mm^3^), Akishima Glass Co., Ltd., Tokyo, Japan) were ultrasonically cleaned with 2-propanol to remove organic molecules from the surfaces, thoroughly rinsed with deionized water, and oven-dried at 70 °C.

### 2.2. Preparation of SWCNT/SiO_2_ Composite Precursor Solution

An SiO_2_ precursor solution (**S_Silica_**) was prepared according to our previous study [13]. A mixed solution of TEOS (1.3 g) and oxalic acid (1.1 g) dissolved in ethanol (10 g) was refluxed for 1 h (Si^4+^ concentration of 0.5 mmol g^−1^).

Ethanol was added to dilute a solution (**S_CNT_**) of the ethanol dispersion of SWCNTs (eDIPS-INK) to adjust the concentration to 0.1 mass%. **S_Silica_** and **S_CNT_** at room temperature were mixed to prepare seven different precursor solutions (C/Si molar ratios of 0.05–0.5) for fabricating the composite thin film. Each mixed solution was mechanically stirred for 1 h. The obtained solutions are denoted as **Sx**, where “**x**” indicates an alphabetical order from “**a**” to “**g**” that corresponds to the C/Si molar ratios of 0.05, 0.1, 0.15, 0.2, 0.3, 0.4, 0.5, respectively (see Table 1).

### 2.3. Fabrication of SWCNT-SiO_2_ Composite Thin Films by UV-Light Irradiation, and Post-Annealing Treatment

Each **Sx** (300 μL) solution was dropped onto a quartz glass substrate and was subsequently spin-coated in double-step mode (1st: 500 rpm for 5 s and 2nd: 2000 rpm for 30 s). The precursor films (**Px**) were obtained after preheating the spin-coated films in a drying oven at 70 °C for 10 min. A germicidal lamp was used to irradiate each **Px** set with a UV light (4 mW cm^−2^ at 254 nm) for 64 h in a clean bench. A digital thermocouple and hygrometer were respectively used to monitor the surface temperature of **Px** and the relative humidity of the clean bench during UV irradiation. The UV-irradiated films on the quartz glass substrate are denoted as **Fx**, corresponding to the applied **Sx** in the spin-coating process. The obtained thin film **Ff** with a C/Si ratio of 0.4 was heat treated at 500 °C for 1 h in an electric furnace to yield **Ff-HT**.

Additionally, the abovementioned procedure was used to obtain a CNT thin film (**F_CNT_**), following spin coating of the **S_CNT_** and preheating. The heat-treated **F_CNT_-HT** film was also obtained following heat treatment of **F_CNT_** at 500 °C for 1 h in an identical furnace.

### 2.4. Electrical and Optical Characterization of Thin Films

A four-probe method employing two multimeters (VOAC7512, Iwatsu and Model 2010 Multimeter, Keithley, Cleveland, OH, USA) and a regulated DC power supply (Model PAB 32-1.2, Kikusui Electronics Corp., Kanagawa, Japan) were used to measure the electrical voltage of **Fa**–**Fg** at 25 °C. Four gold-plated tungsten probes (FELL type, K&S, Washington, DC, USA) were placed at intervals of 1 mm, and a load of 0.1 kg was applied.

A UV-3600 spectrophotometer (Shimadzu, Kyoto, Japan) in double-beam mode with air as a reference was used to measure the transmittance spectra of **Ff** and **Ff-HT** in the 220–2600 nm range. A far-ultraviolet monochromator in single-beam mode (based on KV-200, Bunkohkeiki Co., Ltd., Tokyo, Japan), which was purged with nitrogen gas, was used for measurement in the wavelength range of 130–300 nm; the purging gas was used as a reference.

A MARY-102 ellipsometer was used to measure the refractive indices of **Ff** and **Ff**-**HT** (Five Lab, Kanagawa, Japan) at a wavelength of 632.8 nm and an incident angle of 70.07°. The refractive index was measured at 14 points on the films, and SiO_2_ (refractive index of 1.52) was used as the reference.

A haze meter COH7700 (Nippon Denshoku Industries Co., Ltd., Tokyo, Japan) was used to measure the haze of **Ff** and **Ff**-**HT**. The haze was calculated as the average value from five points on the films, excluding the highest and lowest values.

### 2.5. Structural Characterization of Thin Films

A Raman microspectrometer (LaBRAM HR800, Horiba Ltd., Kyoto, Japan) with a charge-coupled device detector in back-scattering geometry was used to measure the Raman spectra of five films: **Pf**, **Ff**, **Ff**-**HT**, **F_CNT_**, and **F_CNT_-HT**. A Nd-YAG laser (532 nm) with a spot diameter of 1 µm was used as the excitation source at an intensity of 13 mW. The exposure time was changed to determine the optimum conditions for peak detection. The spectra of **Pf**, **Ff**, and **Ff-HT** in the range of 250–2100 cm^−1^ were obtained with an exposure time of 60 s. The spectra of **F_CNT_** and **F_CNT_-HT** were obtained with an exposure time of 30 s. The spectra of **Pf**, **Ff**, and **Ff-HT** in the range of 100–250 cm^−1^ were obtained with an exposure time of 360 s, while the spectra of **F_CNT_** and **F_CNT_-HT** in the identical range were obtained with an exposure time of 180 s.

A Raman peak of the Si substrate at 520.64 cm^−1^ was used for wavelength calibration. A nonlinear least-squares method, which involved a Lorentz function, was used to deconvolute the Raman peaks in the range of 1200–1700 cm^−1^. OriginPro2018b (OriginLab Corporation, Northampton, MA, USA) was used for calculations, and the peak fitting converged with a χ^2^ tolerance value of 1 × 10^−9^.

### 2.6. Surface Morphology, Film Thickness, and Pencil Hardness of Thin Films

Field-emission scanning electron microscopy (FE-SEM) with a JSM-6701F microscope (JEOL Ltd., Tokyo, Japan) at an accelerating voltage of 5 kV was used to determine the surface morphologies of **Pf**, **Ff**, and **F_CNT_**. The average thickness of the SWCNT bundles in **Pf** and **Ff** was calculated from five randomly selected bundles.

Atomic force microscopy (AFM) equipment (OLS4500, OLYMPUS, Tokyo, Japan) was used to obtain three-dimensional (3D) AFM images of **Ff**, **Ff-HT**, **F_CNT_**, and **F_CNT_-HT** by scanning a 2 × 2 μm^2^ area, and the surface roughness was estimated.

Scanning transmission electron microscopy (STEM) and energy dispersive spectroscopy (EDS) in JEM-2100F (JEOL Ltd., Tokyo, Japan) at an accelerating voltage of 200 kV were used to analyze the cross-sectional morphology and elemental distribution of **Ff** and **Ff**–**HT**. The film surface was covered with a carbon film before the center of the sample was cut with a focused ion beam (FIB).

A stylus profilometer (DEKTAK-3, Sloan, Santa Barbara, CA, USA) was used to measure the film thicknesses of **Pf**, **Fx**, and **Ff-HT**. A portion of each precursor film was removed during sample preparation, using ethanol to expose the substrate. The differences at each level between the substrate and the resultant film were measured at five positions for each sample. The film thickness was calculated as the average value, excluding the highest and lowest values. In the case of **Pf**, the measurement was performed two days after the film formed.

A pencil scratch test (the Japanese Industrial Standard (JIS) K5400) was used to evaluate the pencil hardness of **Ff** and **Ff-HT**. The test involved an MJ-PHT pencil hardness meter (Sato Shouji Inc., Kanagawa, Japan) with a 0.75 kg load. Pencils (UNI, Mitsubishi Pencil Co., Ltd., Tokyo, Japan) with various hardness values were standardized in the hardening order from 6B to 9H and were used to scratch the film.

## 3. Results and Discussion

Although an aqueous dispersion of MWCNTs was used in our previous study [13], the precursor solutions (**Sx**) for the SWCNT-silica composite thin film can be prepared by mixing the Si^4+^ complex of oxalic acid in ethanol and an ethanolic dispersion of SWCNTs. The coatability of **Sx** on the quartz glass substrate was excellent and was independent of the SWCNT concentration. The surface temperature of each precursor film during exposure to UV was 20–40 °C, and the humidity in the clean bench was 20–50%. The various properties of the composite film **Ff** with a C/Si ratio of 0.4 are examined in detail in the following sections because this thin film exhibited the lowest electrical resistivity.

### 3.1. Electrical Resistivity of Thin Films and SWCNT Concentration in Precursor

Table 1 lists the electrical resistivities of the composite thin films, **Fa**–**Fg** (thickness: 60–120 nm, Supplementary Materials Figure S1). The volume concentration of SWCNTs was calculated on the assumption that the SWCNTs and SiO_2_ were close packed; therefore, respective densities of 1.3 and 2.2 g cm^−3^ were used [14,15]. Generally, the electrical resistivity of the composite decreased drastically near the percolation threshold, resulting in electrical resistivity at 3.3 vol.%, and reached the lowest value at 12 vol.% (Table 1 and Supplementary Materials Figure S2). The electrical resistivity of **Ff** on the order of 10^−3^ Ω·cm is comparable to that of the ordinary ITO and FTO glass substrates. This low resistivity is associated with the formation of a conductive network unique to CNTs [10,16].

Contrastingly, the lowest resistivity of the SWCNT-silica film was 6.6 Ω·cm; this film was formed by the sol-gel method, in which the maximum CNT concentration was 4 mass% [17]. This concentration corresponds to films **Fb** and **Fc**, which had a proportionally low resistivity. In the case of the present MPM, the resistivity of **Ff** was reduced by as much as three orders of magnitude because the CNT concentration could be further increased. Importantly, the Si^4+^ complex did not interfere with the dispersion of the SWCNTs at high concentrations.

### 3.2. Optical Properties of **Ff** and **Ff-HT**

**Figure 1:**
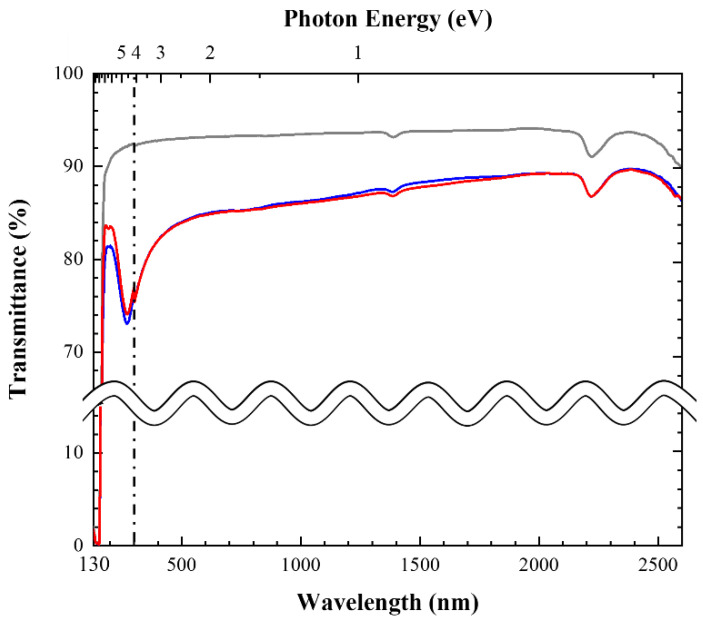
Transmittance spectra of **Ff** (**—**), **Ff-HT** (**—**), and quartz glass substrate (**—**). Spectra were measured in two separate wavelength regions by using different spectrometers and conditions: first region shorter than 300 nm by using KV-200 under N_2_ gas flow and second longer than 300 nm by using UV-3600 in air. The vertical dash-dotted auxiliary line indicates the wavelength at which the spectrometer was changed.

Figure 1 shows the transmittance spectra of **Ff**, **Ff-HT**, and the quartz glass substrate in the range of 130–2600 nm. The transmittance of **Ff** was higher than 80% from 178 nm in the VUV region to the near IR region, and higher than 73% in the range of 220–352 nm. The corresponding value of **Ff-HT** was 1–3% higher than that of **Ff**. Because the quartz glass solely absorbed VUV light, it had the shortest wavelength absorption, and the transmittance consequently decreased because of the substrate thickness. The weak peak at 273 nm (4.5 eV) is due to the π-plasmon absorption of the CNTs, related to the polarization dependence and the optical properties of graphite [18].

The refractive indices of **Ff** and **Ff-HT** were 1.49 and 1.48, respectively, and were comparable to 1.52 for SiO_2_. The haze values of **Ff** and **Ff-HT** were 1.5% and 1.0%, respectively, which indicate the flatness of the film surface (*vide supra*). Notably, current thin films with a low refractive index and haze are much more transparent than ordinary ITO and FTO.

### 3.3. Structural Characterization of **Ff**, **Ff-HT**, **Pf**, **F_CNT_**, and **F_CNT_-HT**

**Figure 2:**
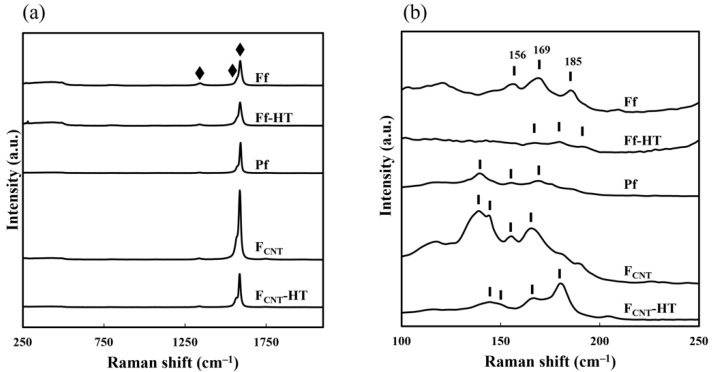
Raman spectra of **Ff**, **Ff-HT**, **Pf**, **F_CNT_**, and **F_CNT_-HT**. (**a**) “♦” indicates peaks attributable to the vibrational modes of CNTs in the range of 250–2100 cm^−1^, (**b**) “**┃**” indicates peaks attributable to the RBM of CNTs in the spectral range of 100–250 cm^−1^.

Figure 2 shows the Raman spectra of **Ff**, **Ff-HT**, **Pf**, **F_CNT_**, and **F_CNT_-HT**. The three peaks at 1340, 1570, and 1590 cm^−1^ in all films can be assigned to the D, G^−^, and G^+^ bands of CNT [19,20]. These three peaks were deconvoluted to evaluate the structural changes of the SWCNTs (Supplementary Materials Figure S3).

The (G^−^ + G^+^)/D ratios of **F_CNT_** and **Pf** were 52 and 42, respectively, suggesting that upon mixing the SWCNTs with the Si^4+^ complex of oxalic acid in ethanol, the number of defect sites in graphite preferentially increased. The (G^−^ + G^+^)/D ratio for **Ff** is 7.1 and ~1/6 of that for **Pf**, indicating a significant increase in the number of defect sites upon UV irradiation. Thus, UV irradiation of **Pf** induced structural changes of the SWCNTs. Despite these significant structural changes, this method is still advantageous for forming highly conductive composites at room temperature.

The (G^−^ + G^+^)/D ratios of **F_CNT_-HT** and **Ff-HT** were respectively 26 and 15, which are respectively half and twice the ratios before heat treatment. Therefore, the number of defect sites in the SWCNTs alone increased than for those embedded in the silica matrix, suggesting that the aforementioned increase in the number of defect sites after UV irradiation might be temporary and that the original graphite ones recovered after heat treatment. Notably, the SiO_2_ matrix plays an important role in preventing degradation of the graphite sites of the SWCNTs during heat treatment.

Multiple peaks in the range of 100–250 cm^−1^, representing the radial breathing mode (RBM) of CNT [20], were found: 139, 156, and 169 cm^−1^ for **Pf**; 156, 169, and 185 cm^−1^ for **Ff**; 167, 179, and 191 cm^−1^ for **Ff-HT**; 139, 144, 156, and 165 cm^−1^ for **F_CNT_**; 146, 152, 167, and 180 cm^−1^ for **F_CNT_-HT**. In comparison with the RBM peaks of **F_CNT_**, those of **Ff** shifted to higher energy, suggesting that dispersion of SiO_2_ in the matrix led to the relaxation of the CNT bundles [21].

### 3.4. Changes in the Film Thickness and Hardness for **Pf** after UV Irradiation and for **Ff** after Heat Treatment

The film thicknesses of **Pf**, **Ff**, and **Ff-HT** were 110, 60, and 70 nm, respectively. Because the as-coated precursor film was too soft, the thickness measurement for **Pf** was performed after keeping the film in the dark for 48 h at 25 °C; nevertheless, the pencil hardness of **Pf** was less than 6B. Comparatively, the pencil hardness of **Ff** and **Ff-HT** was 4H and 9H, respectively. Therefore, UV irradiation of **Pf** at room temperature and heat treatment of **Ff** were both useful for forming robust composite thin films due to the stepwise removal of organic residues in the original precursor film and the UV-irradiated composite thin film, respectively. The film thickness of **Ff-HT** may increase when oxygen atoms are embedded into the Si-O dangling bonds, as observed for an MWCNT composite that we previously prepared [13].

### 3.5. Morphology of Surface and Cross-Section of Thin Films

**Figure 3:**
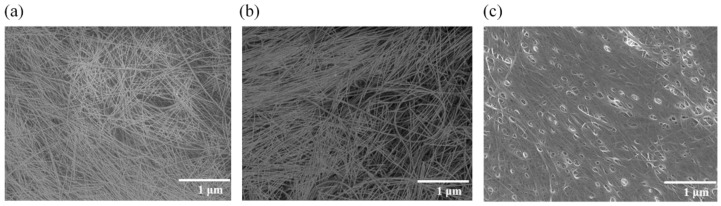
FE-SEM images of (**a**) **Ff**, (**b**) **Pf**, and (**c**) **F_CNT_**.

Figure 3 shows the FE-SEM images of **Ff**, **Pf**, and **F_CNT_**. The SWCNT bundles in **Ff** and **Pf** (Figure 3a,b) are randomly arrayed and the intertwined networks of SWCNT bundles have average diameters of ~16 nm. Comparatively, **F_CNT_**, which was solidified with its dispersant, has many pinholes on its surface (Figure 3c).

**Figure 4:**
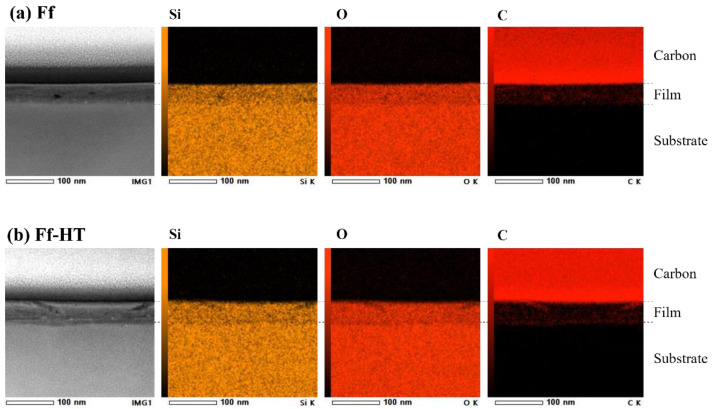
Cross-sectional STEM images and corresponding EDS elemental maps of Si, O, and C signals for (**a**) **Ff** and (**b**) **Ff-HT**.

One bundle contains up to 250 molecules, assuming that one SWCNT molecule is 1 nm in diameter and that adjacent molecules connect directly to form a bundle with a diameter of 16 nm. These bundles with high aspect ratios are arranged horizontally toward the surface and are intertwined. Therefore, it is acceptable that the SWCNT molecules that were dispersed in finer units cannot be observed with FE-SEM. As the Si^4+^ complex photodegraded, the molecular precursor enhanced the contact between the SWCNTs during film formation, resulting in densification of the SiO_2_ matrix and a low electrical resistivity. In fact, the film thickness of **Ff** decreased to almost half that of the precursor film, **Pf**. Comparing the distribution of the SWCNT bundles in **Ff** and **Pf**, it is assumed that densification of the matrix occurred during UV irradiation without aggregation of the SWCNT bundles. From the 3D-AFM images, the surface roughness of **Ff** and **Ff-HT** was 3 nm, which was smaller than 7 and 9 nm for **F_CNT_** and **F_CNT_-HT**, respectively (Supplementary Materials Figure S4). Figure 4 shows the cross-sectional STEM images and corresponding EDS elemental maps of **Ff** and **Ff-HT**. The interface and surfaces were smooth in both cases; there were no cracks or pinholes in the entire film. Importantly, there was little aggregation of the SWCNTs, resulting sufficient dispersion of the carbon atoms.

## 4. Conclusions

SWCNT/SiO_2_ composite precursor solution was prepared by mixing a novel SiO_2_ precursor solution with an ethanol dispersion of SWCNTs. As a result, the precursor film involves the well dispersed SWCNTs in the matrix that consisted of the dried Si complex having oxalic acid as its ligand. By the weak UV-irradiation, the oxalic acid contained as a ligand could be effectively decomposed at room temperature and carbon atoms, originally belonging to oxalic acid, were removed from the film as carbon oxides, producing SiO_2_ matrix. The electrical resistivity of the composite thin film with an SWCNT concentration of 12 vol.% (7.4 mass%) was of the order of 10^−3^ Ω·cm, indicating high transparency over a wide wavelength range spanning the VUV to near IR regions. This resulted from the facile preparation of the precursor solutions via the molecular precursor method, which involved Si^4+^ complex and unprecedentedly high SWCNT content. The current thin film is much more conductive than the MWCNT-SiO_2_ composite thin film. Heat treatment in air hardened the composite thin film without damaging the electrical resistivity and transmittance. The apparent percolation threshold of the SWCNT-SiO_2_ composite thin film was found at an SWCNT volumetric fraction of 3.3 vol%, and this value may be relevant to the formation of SWCNT bundles. This thin film not only has high transmittance in the visible light region, but also has a refractive index equivalent to that of quartz glass. Therefore, the thin film is promising as an alternative material for ITO because it reduces the transmittance of electrodes, associated with light reflection. The composite thin film is sufficiently transparent in the VUV and DUV regions, which will contribute to developing new optoelectronic devices and improving the performance of various devices, including LEDs and solar cells.

## Figures and Tables

**Table 1:** C/Si molar ratio, SWCNT concentration, and electrical resistivity of SWCNT/SiO_2_ composite film.

Composite Film	C/Si Molar Ratio	SWCNT Concentration vol.%	Electrical Resistivity × 10^−3^ Ω·cm
**Fa**	0.05	1.7	>10^6^
**Fb**	0.1	3.3	(2.2 ± 0.4) × 10^3^
**Fc**	0.15	4.8	(5.1 ± 0.6) × 10^3^
**Fd**	0.2	6.3	98 ± 10
**Fe**	0.3	9.2	17 ± 1
**Ff**	0.4	11.9	7.7 ± 0.4
**Fg**	0.5	14.5	10 ± 1

## Data Availability

Not applicable.

## Supplementary Materials

The following are available online at https://www.mdpi.com/article/10.3390/nano11123404/s1, Figure S1: Relationship between the film thickness of the SWCNT/SiO_2_ composite thin films (**Fa**–**Fg**) and their volumetric fraction of SWCNT in each film. A stylus profilometer was used to measure the film thicknesses of **Fx**. Figure S2: Relationship between the electrical resistivity of the SWCNT/SiO_2_ composite thin films (**Fb**–**Fg**) and their volumetric fraction of SWCNT in each film. The electrical resistivity of **Fb**–**Fg** at 25 °C was determined using four-probe method and film thickness. The plot of **Fa** was removed from the figure because the value is >10^3^ Ω·cm. Figure S3: Deconvoluted Raman peaks of the obtained thin films, (a) **Ff**, (b) **Ff-HT**, (c) **Pf**, (d), **F_CNT_**, and (e) **F_CNT_-HT**, along with each original spectrum in the range of 1200–1700 cm^−1^. The thick line indicates the observed spectra, while three lines, (---), (_•••_), and (—) indicate the theoretically fitted curves assignable the D, G^−^, and G^+^ bands, respectively. All the calculated curves completely overlapped the corresponding observed curves. Figure S4: Three-dimensional (3D) AFM images of (a) **Ff**, (b) **Ff-HT**, (c) **F_CNT_**, and (d) **F_CNT_-HT** obtained by scanning each 2 × 2 μm^2^ area.

## References

[1] Chopra K.L., Major S., Pandya D.K. (1983). Transparent conductors—A status review. Thin Solid Film..

[2] Kang M., Kim I., Chu M., Kim S.W., Ryu J.-W. (2011). Optical Properties of Sputtered Indium-tin-oxide Thin Films. J. Korean Phys. Soc..

[3] Iijima S., Ichihashi T. (1993). Single-shell carbon nanotubes of 1-nm diameter. Nature.

[4] Grunlan J.C., Mehrabi A.R., Bannon M.V., Bahr J.L. (2004). Water-based single-walled-nanotube-filled polymer composite with an exceptionally low percolation threshold. Adv. Mater..

[5] Ata S., Mizuno T., Nishizawa A., Subramaniam C., Futaba D.N., Hata K. (2014). Influence of matching solubility parameter of polymer matrix and CNT on electrical conductivity of CNT/rubber composite. Sci. Rep..

[6] Buldum A., Lu J.P. (2001). Contact resistance between carbon nanotubes. Phys. Rev. B.

[7] Nirmalraj P.N., Lyons P.E., De S., Coleman J.N., Boland J.J. (2009). Electrical connectivity in single-walled carbon nanotube networks. Nano Lett..

[8] Djelad H., Benyoucef A., Morallón E., Montilla F. (2020). Reactive insertion of PEDOT-PSS in SWCNT@silica composites and its electrochemical performance. Materials.

[9] Tsetseris L., Pantelides S.T. (2006). Encapsulation of floating carbon nanotubes in SiO_2_. Phys. Rev. Lett..

[10] He J., Chen J., Shi L., Li Q., Lu W., Qu S., Qiu W., Zhou G. (2019). Fabrication of thermally robust carbon nanotube (CNT)/SiO_2_ composite films and their high-temperature mechanical properties. Carbon.

[11] de Andrade M.J., Weibel A., Laurent C., Roth S., Bergmann C.P., Estournès C., Peigney A. (2009). Electrical conductive double-walled carbon nanotubes—Silica glass nanocomposites prepared by the sol-gel process and spark plasma sintering. Scr. Mater..

[12] Nagai H., Sato M. (2018). The Science of Molecular Precursor Method. Advanced Coating Materials.

[13] Nagai H., Ogawa N., Sato M. (2021). Deep-Ultraviolet Transparent Conductive MWCNT/SiO_2_ Composite Thin Film Fabricated by UV Irradiation at Ambient Temperature onto Spin-Coated Molecular Precursor Film. Nanomaterials.

[14] Suzuki S., Sato S., Kakita K. (2008). Analysis of SiO_2_ Films on Si Substrate by GD-OES Depth Profiling and GIXR Measurements. J. Surf. Anal..

[15] Jamali V., Mirri F., Biggers E.G., Pinnick R.A., Liberman L., Cohen Y., Talmon Y., Mackintosh F.C., Van Der Schoot P., Pasquali M. (2021). Enhanced ordering in length-polydisperse carbon nanotube solutions at high concentrations as revealed by small angle X-ray scattering. Soft Matter.

[16] Zhan G.D., Kuntz J.D., Garay J.E., Mukherjee A.K. (2003). Electrical properties of nanoceramics reinforced with ropes of single-walled carbon nanotubes. Appl. Phys. Lett..

[17] De Andrade M.J., Lima M.D., Stein L., Bergmann C.P., Roth S. (2007). Single-walled carbon nanotube silica composites obtained by an inorganic sol-gel route. Phys. Status Solidi.

[18] Murakami Y., Einarsson E., Edamura T., Maruyama S. (2005). Polarization dependence of the optical absorption of single-walled carbon nanotubes. Phys. Rev. Lett..

[19] Jorio A., Filho A.G.S., Dresselhaus G., Dresselhaus M.S., Swan A.K., Ünlü M.S., Goldberg B.B., Pimenta M.A., Hafner J.H., Lieber C.M. (2002). G-band resonant Raman study of 62 isolated single-wall carbon nanotubes. Phys. Rev. B.

[20] Osswald S., Flahaut E., Gogotsi Y. (2006). In situ raman spectroscopy study of oxidation of double- and single-wall carbon nanotubes. Chem. Mater..

[21] Dieckmann G.R., Dalton A.B., Johnson P.A., Razal J., Chen J., Giordano G.M., Muñoz E., Musselman I.H., Baughman R.H., Draper R.K. (2003). Controlled assembly of carbon nanotubes by designed amphiphilic peptide helices. J. Am. Chem. Soc..

