# Supplementary material for: Fabrication of Transparent and Conductive SWCNT/SiO2 Composite Thin-Film by Photo-Irradiation of Molecular Precursor Films
Source: Nanomaterials (Basel). 2021 Dec 16;11(12):3404. doi: 10.3390/nano11123404 (PMC8707605; doi:10.3390/nano11123404)
Supplement: Supplementary file 1 [file nanomaterials-11-03404-s001.zip › nanomaterials-1471955-supplementary.pdf]

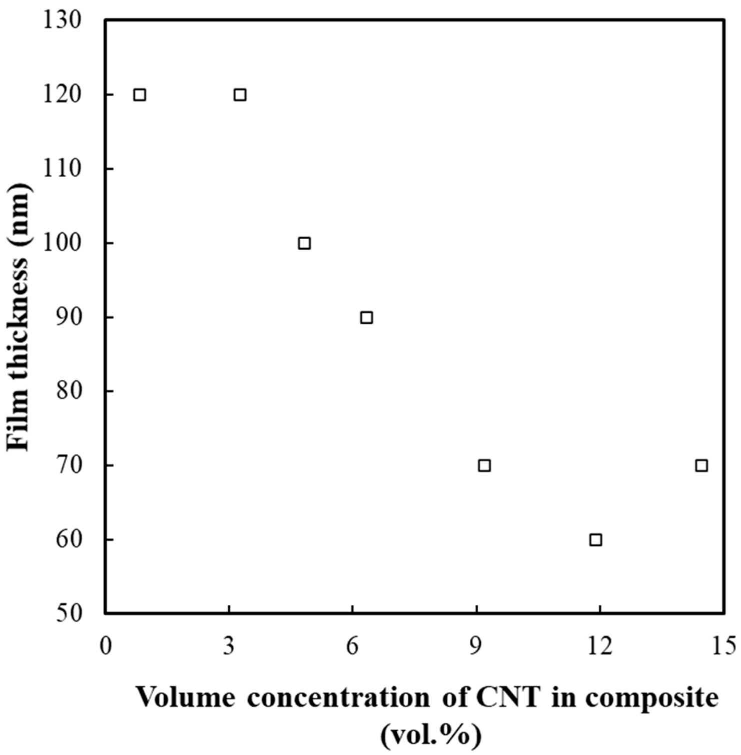

Supplementary Figure S1. Relationship between the film thickness of the SWCNT/SiO_2 composite thin films (**Fa**−**Fg**) and their volumetric fraction of SWCNT in each film. A stylus profilometer was used to measure the film thicknesses of **Fx**.

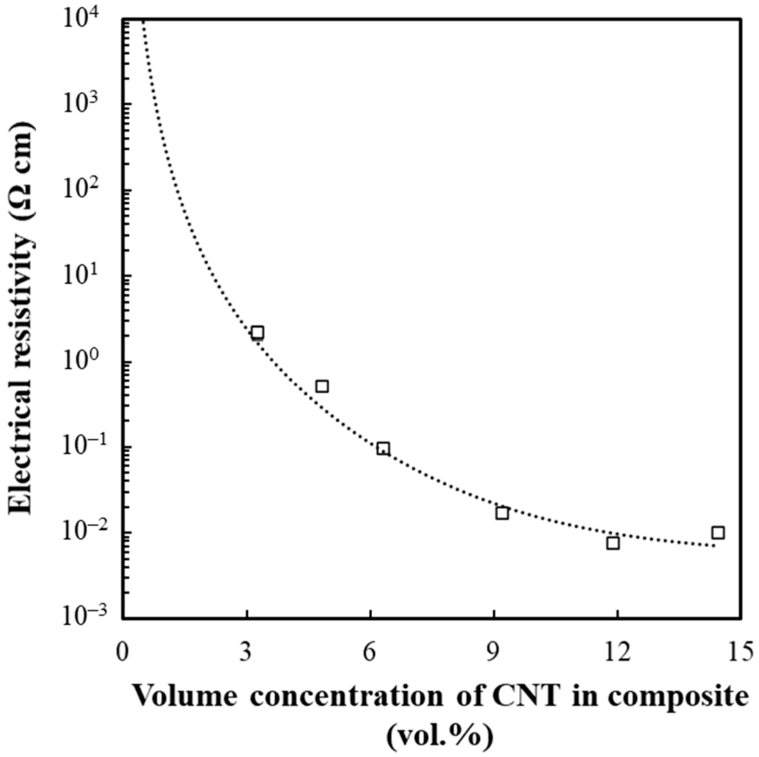

Supplementary Figure S2. Relationship between the electrical resistivity of the SWCNT/SiO_2 composite thin films (**Fb**−**Fg**) and their volumetric fraction of SWCNT in each film. The electrical resistivity of **Fb**–**Fg** at 25 °C was determined using four-probe method and film thickness. The plot of **Fa** was removed from the figure because the value is >10^3 Ω cm.

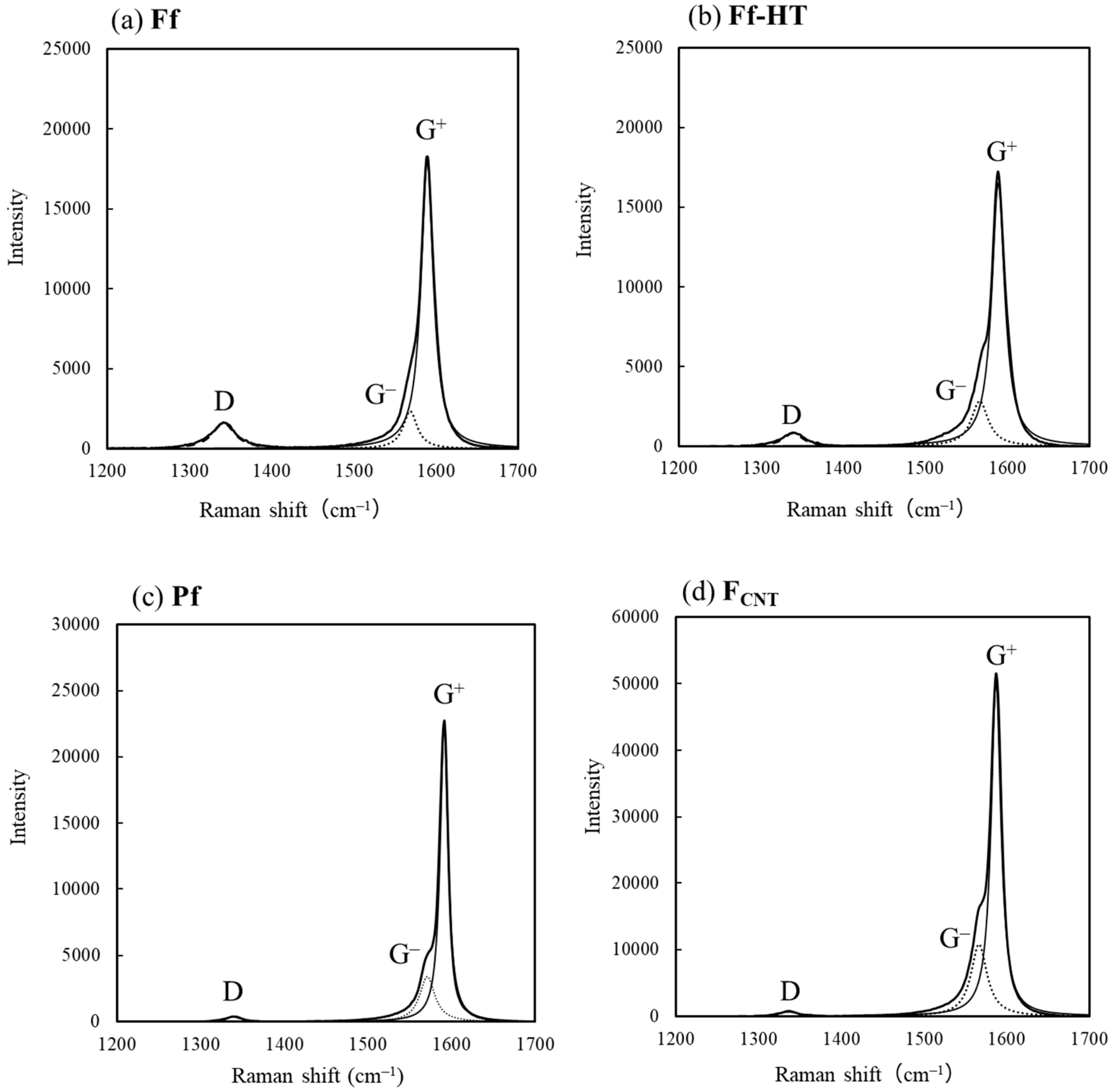
(a) Ff
Intensity
Raman shift（cm⁻¹）
D
G⁻
G⁺
(b) Ff-HT
Intensity
Raman shift（cm⁻¹）
D
G⁻
G⁺
(c) Pf
Intensity
Raman shift (cm⁻¹)
D
G⁻
G⁺
(d) F_CNT
Intensity
Raman shift（cm⁻¹）
D
G⁻
G⁺

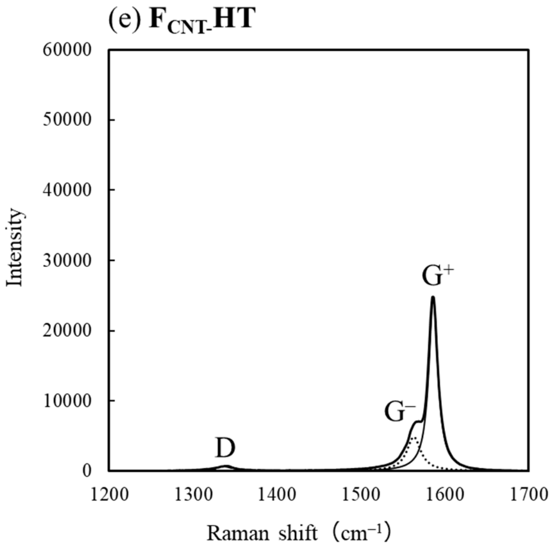

Supplementary Figure S3. Deconvoluted Raman peaks of the obtained thin films, (a) **Ff**, (b) **Ff-HT**, (c) **Pf**, (d), $\mathbf{F_{CNT}}$, and (e) $\mathbf{F_{CNT}}$**-HT**, along with each original spectrum in the range of 1200-1700 cm^{-1}. The thick line indicates the observed spectra, while three lines, (---), (•••), and (—) indicate the theoretically fitted curves assignable the D, G^{-}, and G^{+} bands, respectively. All the calculated curves completely overlapped the corresponding observed curves.

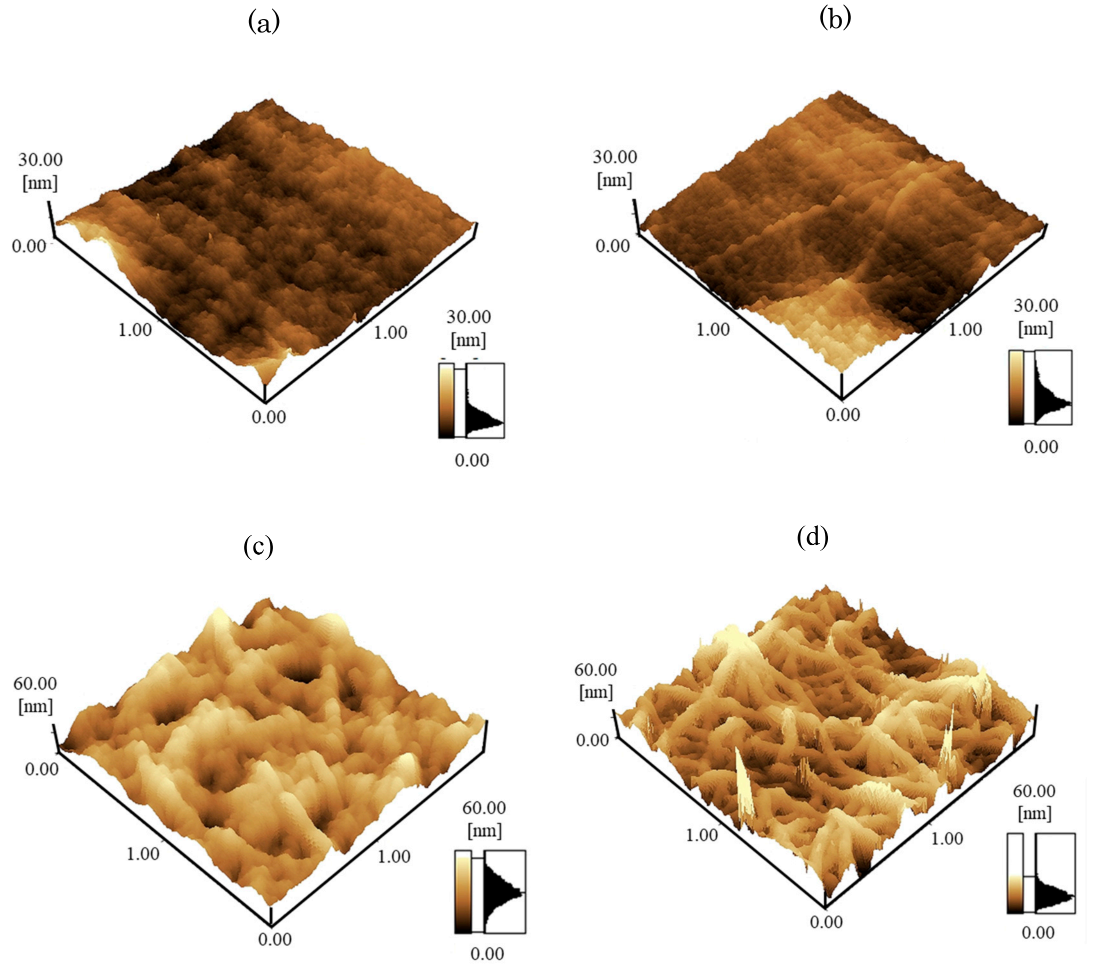

Supplementary Figure S4. Three-dimensional (3D) AFM images of (a) **Ff**, (b) **Ff-HT**, (c) $\mathbf{F_{CNT}}$, and (d) $\mathbf{F_{CNT}}$**-HT** obtained by scanning each 2 × 2 μm^2 area.
